# Transcriptome and metabolome analysis reveal candidate genes and biochemicals involved in tea geometrid defense in *Camellia sinensis*

**DOI:** 10.1371/journal.pone.0201670

**Published:** 2018-08-01

**Authors:** Wei-Wei Wang, Chao Zheng, Wan-Jun Hao, Chun-Lei Ma, Jian-Qiang Ma, De-Jiang Ni, Liang Chen

**Affiliations:** 1 Key Laboratory of Tea Biology and Resources Utilization, Ministry of Agriculture, National Center for Tea Improvement, Tea Research Institute of the Chinese Academy of Agricultural Sciences, Hangzhou, China; 2 Key Laboratory of Horticulture Plant Biology, College of Horticulture and Forestry Sciences, Huazhong Agricultural University, Wuhan, China; ICAR - National Research Center on Plant Biotechnology, INDIA

## Abstract

Tea plant (*Camellia sinensis* (L) O. Kuntze) respond to herbivore attack through large changes in defense related metabolism and gene expression. *Ectropis oblique* (Prout) is one of the most devastating insects that feed on tea leaves and tender buds, which can cause severe production loss and deteriorate the quality of tea. To elucidate the biochemicals and molecular mechanism of defense against tea geometrid (TG), transcriptome and metabolome of TG interaction with susceptible (SG) and resistance (RG) tea genotypes were analyzed by using UPLC-Q-TOF-MS, GC-MS, and RNA-seq technologies. This revealed that jasmonic acid was highly induced in RG, following a plethora of secondary metabolites involved in defense against TG could be induced by jasmonic acid signaling pathway. However, the constitutively present of salicylic acid in SG might be a suppressor of jasmonate signaling and thus misdirect tea plants against TG. Furthermore, flavonoids and terpenoids biosynthesis pathways were highly activated in RG to constitute the chemical barrier on TG feeding behavior. In contrast, fructose and theanine, which can act as feeding stimulants were observed to highly accumulate in SG. Being present in the major hub, 39 transcription factors or protein kinases among putative candidates were identified as master regulators from protein-protein interaction network analysis. Together, the current study provides a comprehensive gene expression and metabolite profiles, which can shed new insights into the molecular mechanism of tea defense against TG. The candidate genes and specific metabolites identified in the present study can serve as a valuable resource for unraveling the possible defense mechanism of plants against various biotic stresses.

## Introduction

Plants are sessile organisms and continuously challenged by a wide variety of abiotic and biotic stress factors in their natural habitat. In co-evolution, plants have developed various highly dynamic and variable defense strategies at the molecular, biochemical, and morphological level in response to herbivore insects [1]. In higher plants, the partially known molecular mechanisms in response to herbivore insect attack start with the recognition of defense elicitors (e.g., fatty acid-amino acid conjugates (FACs) and *β*-glucosidase) in insect oral secretions and signals from mechanically injured plant cells. The recognition of these elicitors or signals induces convergent intracellular signaling pathways, such as calcium ion signaling (Ca^2+^-dependent protein kinases, CPKs), phosphorylation cascades (mitogen-activated protein kinases, MAPKs) cascades, leucine-rich repeat receptor-like kinases (LRR-RLKs), and hormonal signaling (e.g., salicylic acid (SA) and jasmonic acid (JA)) in plant cells, which ultimately result in the reprogramming of the transcriptome [1,2]. After signal processing, plants produce specialized morphological structures (e.g., thorns, trichomes, spines, hairs, and thicker leaves) and secondary metabolites (e.g., flavonoids, anthocyanins, alkaloids, and terpenoids) and proteins (lipoxygenases and peroxidases) that have antinutritional repellent, and/or toxic effects on the herbivore insects [1,3].

Tea plant (*Camellia sinensis* (L) O. Kuntze) is one of the most popular non-alcoholic beverage crops worldwide. Its young leaves are processed to prepare "tea", which is widely consumed by millions of people for its biologically active polyphenols, catechins, flavanones, vitamins and medicinal properties [4]. Just like other plants, being sessile in nature, tea plants are continuously exposed to biotic invasions, and a large percentage of yield loss is cost by herbivorous feeding. The tea geometrid (*Ectropis obliqua* (Prout), TG) is one of the most prevalent and devastating chewing pests in tea plantations, whose larvae can cause severe production loss and degradation of tea quality [5]. To guarantee the safety and quality of tea, insecticides and chemical treatments are generally avoided. In this context, the underlying mechanisms of plant-insect interactions and the identification of new ways to improve plant resistance have been considered as an important field of research.

Nowadays, high-throughput omics techniques (e.g. phenomic, ionomic, metabolomic, transcriptomic, proteomic, genomic, etc.) have been extensively employed by plant biologists in their research on plant-insect interactions [6]. Since transcriptional reprogramming underlies many plant defense responses, transcriptomic analyses of responses to TG and other herbivore insects have been conducted using several plant species [7–9]. In our previous study, we compared the transcriptomes of tea plants subject to TG damage with mechanically damaged and control treatment [7]. Our results demonstrated that terpenoid synthesis, phenylpropanoid biosynthesis, JA and brassinosteroid signaling pathways were highly activated in response to TG attack. In addition, using another tea plant cultivar (*Camellia sinensis* cv. Shuchazao), Wang *et al*. also employed RNA-Seq technology to compare the differential gene expression profiles between the TG damaged tea plants and undamaged control. They revealed the plant secondary metabolites such as caffeine, phenylpropanoid, and herbivore-induced plant volatiles (HIPVs) biosynthesis-related genes were differentially regulated, which may play a crucial role in defense tea plants against TG [9]. In fact, different plant species or different ecotypes of the same species may react differently to the same insect species [10–12]. Comparative approaches, therefore, should be employed on the study of plant-insect relationship.

Recent developments in high-throughput metabolite identification technologies such as gas chromatography-mass spectrometry (GC-MS), liquid chromatography-mass spectrometry (LC-MS), ultra-performance liquid chromatography-tandem mass spectrometry (UPLC-MS), and nuclear magnetic resonance (NMR) have greatly accelerated the field of plant metabolomics research [13]. By applying NMR-based metabolomics, the metabolome profiles of thrips-resistant and -susceptible chrysanthemums can be differentiated by higher amounts of two phenylpropanoids (i.e., chlorogenic acid and feruloyl quinic acid) [14]. Jansen *et al*. monitored metabolic changes in the *B*. *oleracea*-*P*. *rapae* interaction using UPLC-MS, phenylpropanoids were identified as "metabolic interface" that were present in the insect and induced in plant tissue upon feeding [15]. Therefore, RNA-seq and metabolomic based technology on its own can provide novel and valuable information about plant-insect interactions, but data from several approaches should be integrated together to link phenotype and genotype. Currently, chemical ecology of tea plant-insect interaction is less clearly understood and the interactive effects of transcriptomic and metabolomic plant responses to TG feeding have not been investigated so far.

To take a further step toward understanding plant defense responses to TG, the global transcriptome and metabolome profiles were examined for the first time during TG attack in RG and SG. Comparative metabolome and transcriptome analysis permitted us to identify putative metabolites, genes and their interactions in defense pathways during TG attack. Comprehensive efforts of the current study shed a new light on highly variable insect-plant interactions and would thus accelerate genetic improvement of tea and other perennial crops.

## Materials and methods

### Plant materials and stress treatments

Tea plant (*Camellia sinensis*) accessions namely "Pingyang Tezao" and "Shangmeizhou", known source of susceptible (SG) and resistance (RG) response against *E*. *oblique*, were used to explore the possible defense mechanism of tea plant against TG attack. Four-year-old tea plants were grown under a 16-h light (25°C)/8-h dark (20°C) photoperiod in the greenhouse of China National Germplasm Hangzhou Tea Repository for 2 weeks. For insect feeding treatment, twenty 4th instar larvae were starved for 8 h and distributed evenly on the new shoot (one bud and two leaves) of the tea plants, and when approximately 25–35% the leaves were consumed, TGs were removed. Tea leaves from undamaged plants were used as control (CK). Samples were collected at 6 h post-feeding treatment, three and eight biological replicates were harvested from each group of samples for RNA-seq and metabolomic analyses, respectively. All the samples were frozen in liquid nitrogen and stored at -80°C for further use.

### Illumina sequencing and *de novo* assembly

Total RNA was isolated from each leaf sample using the RNeasy Plant Mini Kit (QIAGEN, USA) according to the manufacturer’s protocol. cDNA libraries were constructed with 1.5 μg of total RNA per sample using NEBNext Ultra RNA Library Prep Kit for Illumina (NEB, USA), and the library quality was checked on the Agilent Bioanalyzer 2100 system. After cluster generation, the library preparations were used for 150 bp paired-end sequencing on an Illumina Hiseq 4000 platform. ~704 million high-quality reads were pooled from Illumina sequencing of each of the 6 samples (three biological replicates for RG and SG) and were then assembled into unigenes using Trinity software (v2.4.0) [16].

### Sequence annotation

Gene function was annotated based on the following databases: Nt (NCBI non-redundant nucleotide sequences); Nr (NCBI non-redundant protein sequences); Pfam (Protein family); Swiss-prot (A manually annotated and reviewed protein sequence database); KOG (euKaryotic Ortholog Groups); GO (Gene Ontology); KO (KEGG Ortholog database). All the unigenes were searched against Nr, Nt, Swiss-prot, KO and KOG databases with BLAST *E-*value < 1E-5. GO functional classification was performed using Blast2GO program [17].

### Differential gene expression, GO and KEGG enrichment analysis

Read count normalization and differential expression analysis was implemented by the DESeq2 R package (v3.1) [15]. Genes with an adjusted *P*-value < 0.05 were assigned as differentially expressed. GO and KEGG enrichment analysis of the differential expressed genes (DEGs) was performed using the GOseq R packages [18] and KOBAS software (KOBAS, Surrey, UK) [19], respectively.

### Protein-protein interaction network analysis

To further identify the putative key genes for producing plants resistant to TG, top 2,000 up-regulated genes (RG vs. SG) were mapped to protein-protein interaction (PPI) network of *A*. *thaliana* in STRING database [20]. The network was visualized by using Cytoscape software (v3.6) [21].

### qRT-PCR validation for DEGs

To verify the accuracy of RNA-Seq results, 6 DEGs (i.e., *LDOX*, *MYB308*, *WRKY75*, *PAL*, *LOX2*.*1*, and *TPS21*) were selected for qRT-PCR analysis. qRT-PCR was performed using KAPA SYBR FAST qPCR Kit (Kapa Biosystems, MA, USA) and run on Applied Biosystems 7500 Sequence Detection System (Carlsbad, CA, USA) under the following parameters: 95°C for 3 min, 40 cycles of 95°C for 3 s, 60°C for 34 s. Triplicates of each reaction were performed, GAPDH sequence was used as internal control for normalization. Relative fold changes were calculated using 2^−ΔΔCT^ method [22]. Primer details for RT-PCR are given in S1 Table.

### UPLC-Q-TOF-MS and GC-MS based metabolomic analyses

In total, 32 samples (eight biological replicates for each treatment (i.e., TG feeding and control) and genotype (i.e., SG and RG)) were subjected to metabolomic analyses based on ultra-performance liquid chromatography quadrupole time of flight mass spectrometry (UPLC-Q-TOF-MS) and gas chromatography-mass spectrometry (GC-MS). Plant samples (100 mg) were extracted with 500 μL of methanol/water (3:1, v/v) solvent as described in our previous study [23]. For metabolomic analysis based on UPLC-Q-TOF-MS, 2 μL supernatant was injected into Agilent 6530 Accurate-Mass Q-TOF LC/MS (Agilent, USA) and separated with an HSS T3 column. Agilent MassHunter Qualitative Analysis software (vB.06.00) and XCMS [24] were employed for data preprocessing. For metabolomic analysis based on GC-MS, 1 μL derivatized sample was injected in split-injection mode into the DB-5 capillary column with a split ratio of 30:1 and separated by Agilent 7890A-5975C GC-MS system (Agilent, USA). Data were preprocessed by using the ChromaTOF software (v4.34). Preprocessed datasets of UPLC-Q-TOF-MS and GC-MS were exported to SIMCA-P software (v14.1) for multivariate data analyses. Unsupervised principal component analysis (PCA) and supervised projection to latent structure-discriminant analysis (PLS-DA) were carried out to classification and discrimination between the treatments. In addition, student’s *t*-test was performed by using R statistical toolbox (R 3.3.0, www.r-project.org). Metabolites with VIP > 1.0 and *P*-value < 0.05 were considered as differentially expressed metabolites (DEMs) between samples.

### Accession code

RNA-Seq read data were deposited to the BIGD (BIG Data Center, http://bigd.big.ac.cn/) under accession number CRA000859.

## Results

### Transcriptome sequencing

To dissect the molecular mechanism of tea plants in response to TG attack, six RNA-Seq libraries of three biological replicates for RG and SG were prepared and then paired-end sequenced. After discarding the low-quality raw reads, 16.96G to 18.83G clean data were generated (S2 Table). A total of 704,798,092 high-quality reads were used for a *de novo* assembly of the reference transcriptome. In total, 543,683 transcripts, and 342,961 non-redundant unigenes with an N50 of 834 bp and an average length of 594 bp were achieved (S3 Table). We then searched these unigenes against seven public databases, including KOG, KEGG, GO, Nr, Nt, Swiss-prot and Pfam for identifying homologous sequences. A total of 156,440 (45.61%) unigenes were annotated in at least one databases, and the distributions of in KEGG and GO functional categories and the detailed annotation information were shown and listed in S1 Fig and S4 Table, respectively.

### Differentially express and enrichment analysis of genes respond to *E*. *oblique* attack

The average Pearson correlation coefficients between replicates were 0.843 and 0.828 for RG and SG, respectively (S2 Fig). This indicates that the variations we observed were mostly related to the differences in genetic background. To examine the molecular differences between RG and SG respond to TG attack, the DESeq2 R package was used to identify significantly DEGs. As a result, 14,260 DEGs (adjust *P*-value < 0.05) were obtained, of which 7,587 were up-regulated and 6,673 were down-regulated. The large discrepancy in the transcriptome profiles between two cultivars suggested that RG may develop some distinct response mechanism during TG attack. To test the accuracy of our RNA-Seq data, six DEGs were validated with qRT-PCR, and we found the data from both shared similar trends (S3 Fig).

**Fig 1:**
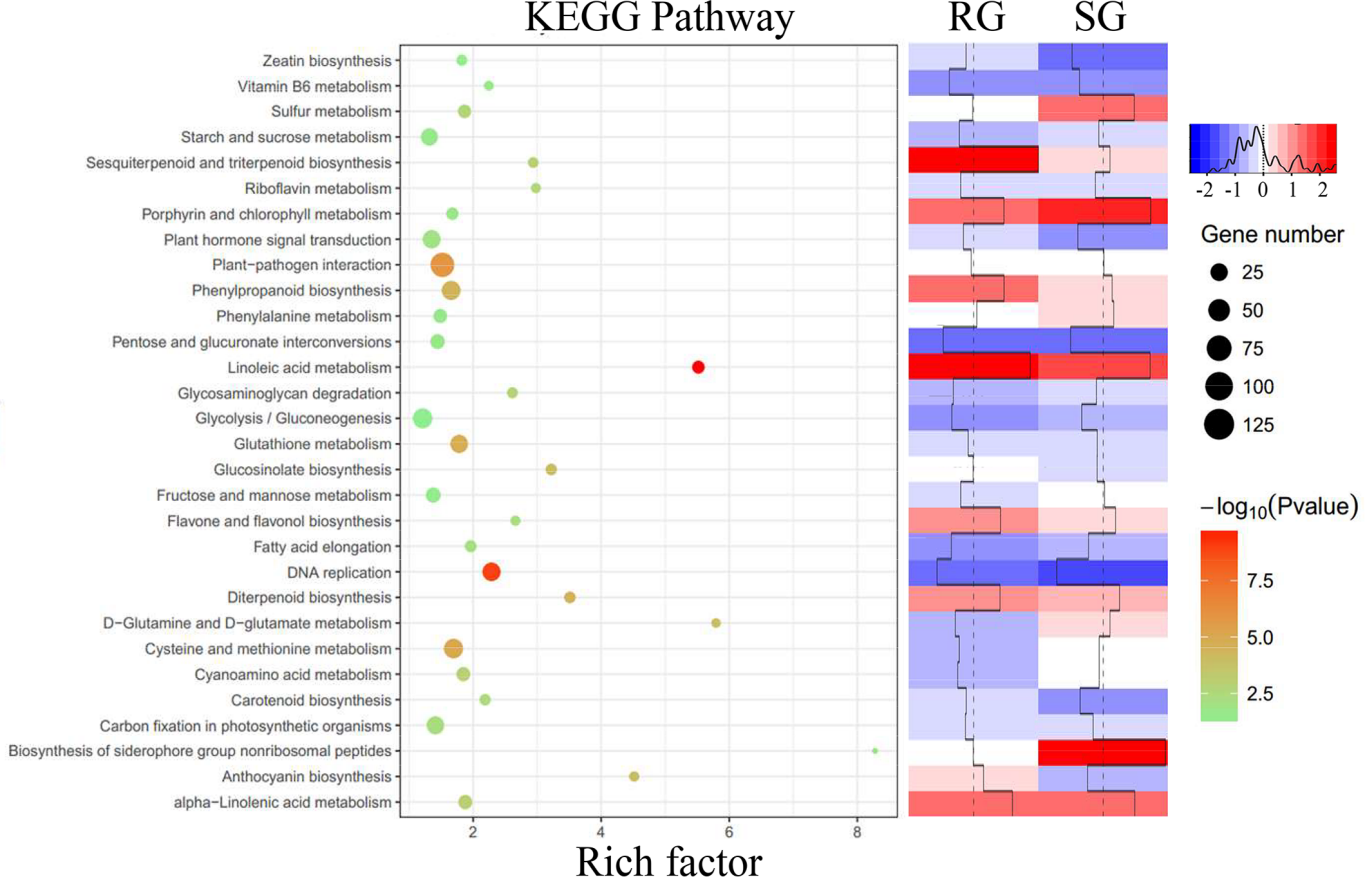
KEGG pathway analysis of differentially expressed genes. The color and size of the dots in the scatterplot represent the range of the negative log10-transformed *P*-value and the gene number, respectively. The heat map on the right showing the overall expression levels of enriched pathways.

We then performed KEGG and GO enrichment analysis to explore the relevant pathways and biological functions that were activated to against TG attack. KEGG pathway analysis revealed that carbohydrate (starch and sucrose, fructose and mannose metabolism) and amino acid (D-glutamine and D-glutamate, cysteine and methionine) metabolism related pathways were more activated in SG. On the other hand, phenylpropanoid (anthocyanin, phenylpropanoid, flavone, and flavonol biosynthesis) and terpenoid biosynthesis (diterpenoid, sesquiterpenoid, and triterpenoid biosynthesis) related pathways were more highly enriched and shown a higher expression level in RG (Fig 1). We then detailed investigated these pathways, and we found genes encoding phenylalanine ammonia-lyase (*PAL*) and leucoanthocyanidin dioxygenase (*LDOX*), which involved in a rate-limiting step of phenolic and anthocyanin biosynthesis, respectively, were significantly induced in RG (S5 Table). In addition, most of the (7 out of 10) terpene synthases genes (e.g., *TPS03*, *TPS04*, and *TPS21*), which catalyze key steps in the formation of terpenoids, were also more induced in RG (S5 Table).

**Fig 2:**
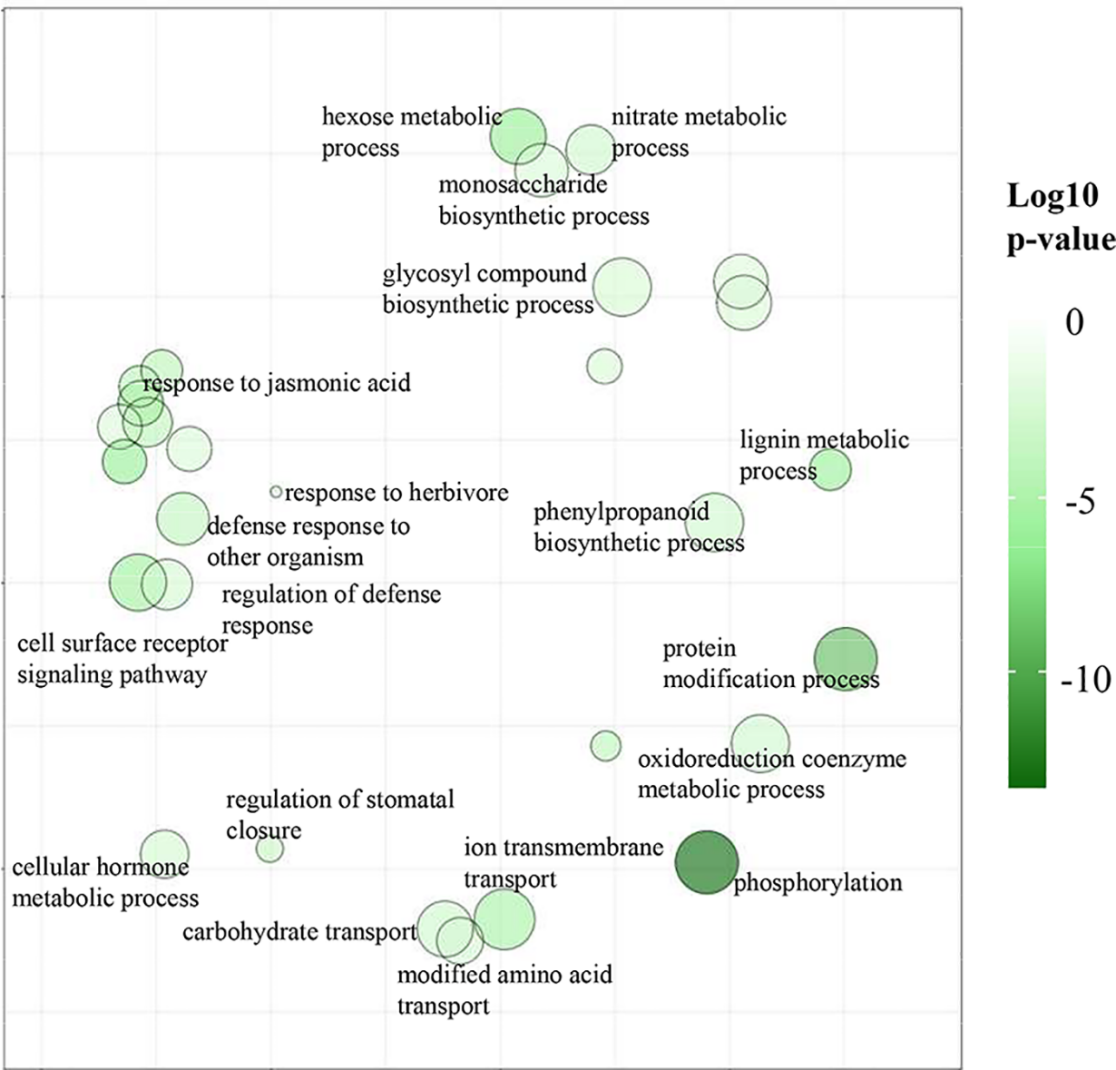
GO enrichment of the genes that showed a higher expression level in RG. The color of the dots in the scatterplot represents the range of the log10-transformed *P*-value.

We used ReviGO tool to collectively visualize the 31 significant enriched GO terms (*P*-value < 0.05 FDR BH corrected) for 7,587 up-regulated genes. And we found that these genes were mainly related to lipid modification, cell surface receptor signaling pathway, xylem development, and transport (ion transmembrane transport, carbohydrate transport, and modified amino acid transport), especially involved in phosphorylation and metabolic process (hexose, oxidoreduction coenzyme, lignin, phenylpropanoid, nitrate and isoprenoid metabolic). Furthermore, several defenses (e.g., regulation of stomatal closure, response to herbivore, and defense response to other organisms) and phytohormone signaling (e.g., response to abscisic acid, cytokinin, jasmonic acid) related GO term, as expected, were also significantly enriched (Fig 2 and S6 Table).

### Protein-protein interactions against TG attack

**Fig 3:**
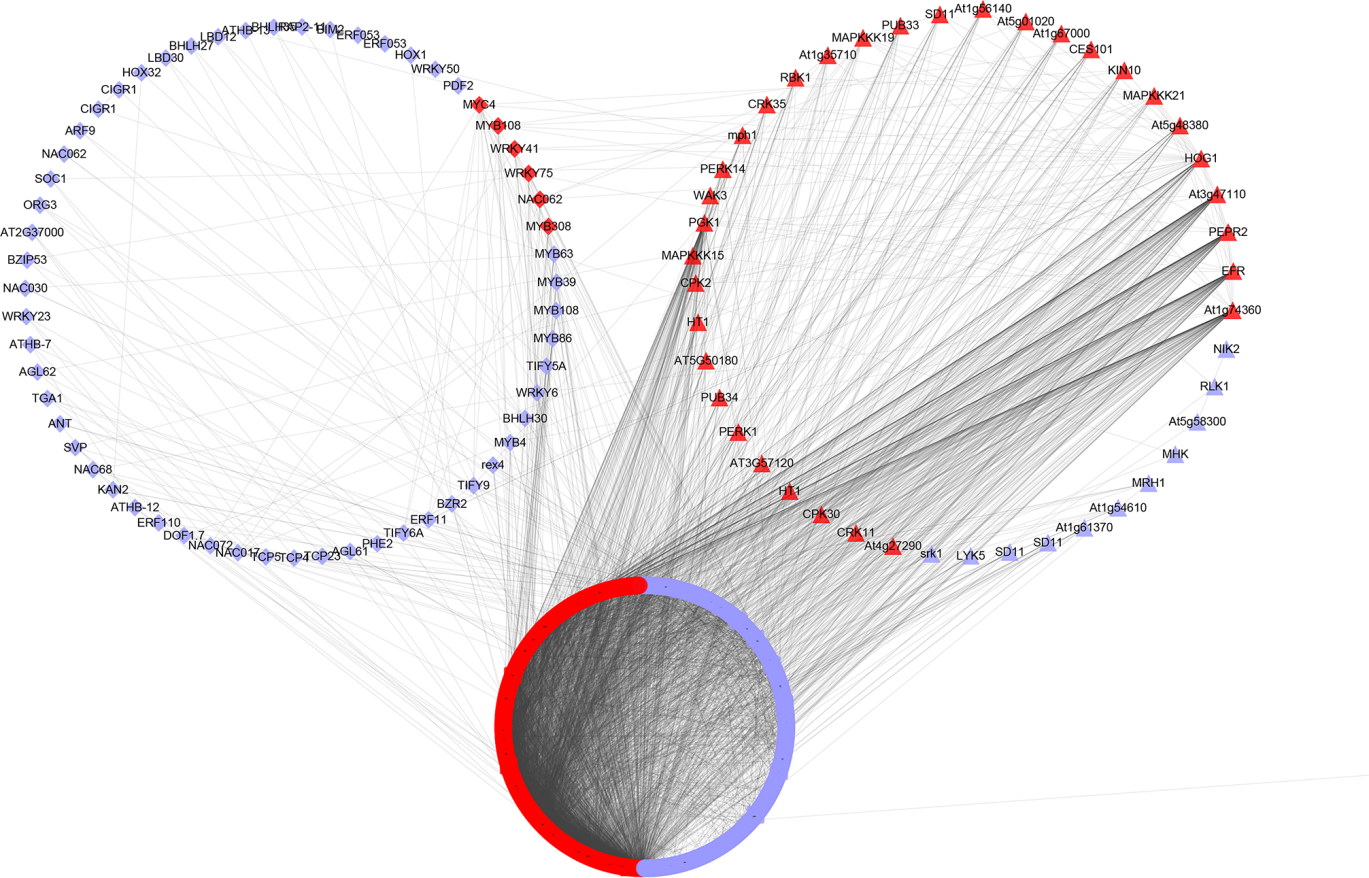
Protein-protein interaction network analysis for the top 2000 genes which showed a higher expression level in RG. This interaction network for the top 2000 genes which showed a higher expression level in RG was created using STRING. Unconnected genes were removed, and the network was visualized in Cytoscape. The diamond nodes represent TFs, the triangle nodes represent PKs, and the TFs or PKs with more than 10 edges were indicated in red color.

For identification of master regulators involved in TG defense, we assigned top 2,000 genes that showed a higher expression level in RG to predetermined protein-protein interaction (PPI) network of *A*. *thaliana* in STRING database [20]. As a result, 997 genes were successfully mapped, among which 103 TFs (59) or PKs (44) were found to be interacting with 894 nodes and 24,294 edges. An average number of undirected neighbors in the network for each gene was 24.367. Those TFs (6) or PKs (33) with more than 10 edges were regarded as controlling hubs against TG attack. Furthermore, PPI network analysis revealed that these candidate TFs, including MYB308/108, WRKY41/75, NAC062, and MYC4, interacting with 61 other genes, and these candidate PKs which were belonging to LRR-RLK, Ser/Thr kinase, CDPK, and MAPK family with 271 other genes; thus, can be potential candidates for enhancing plant resistance to TG (Fig 3 and S7 Table).

### Metabolic analysis

**Fig 4:**
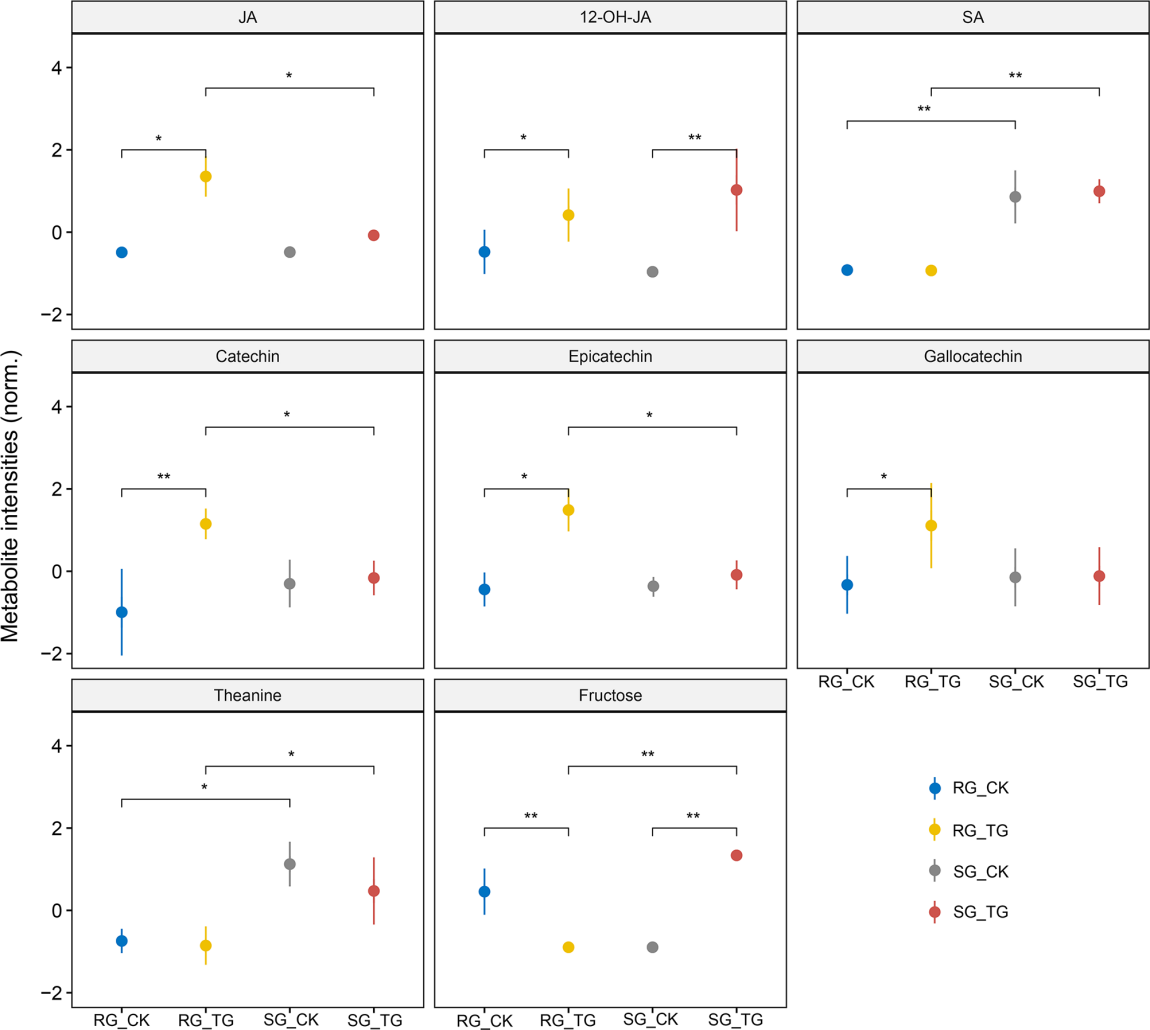
Relative intensity of putative metabolites that influence TG resistance in RG and SG. Mean expression values of metabolite intensities with their standard error bars from eight biological replicates are represented. The asterisks indicate significant differences between different samples ("*" means *P* < 0.05; "**" means *P* < 0.01).

By UPLC-Q-TOF-MS and GC-MS analysis, we detected 4,732 metabolite features during TG infestation in two cultivars. PCA of metabolite data revealed a clear separation between different treatments and cultivars (LC-MS: PC1(27.8%), PC2 (8.5%); GC-MS: PC1(26.1%), PC2 (16.5%); S4 Fig). PLS-DA modeling and student’s *t*-test were employed to determine significantly changed metabolites (VIP > 1, *P* < 0.05). As a result, 75 and 74 differentially expressed metabolites (DEMs) were identified in RG and SG, respectively. These DEMs included flavonoids, amino acids and their derivatives, fatty acids, nucleic acids and their derivatives (S8 Table). In both cultivars, metabolites involved in the flavonoid pathway are highly enriched during TG attack when compared with control plants. On closer analysis, it was found that the type of flavonoid products was totally different. For example, epicatechin (EC), gallocatechin (GC) and catechin (C), and leucocyanidin were uniquely increased their level in RG, whilst kaempferol, delphinidin, quercetin, proanthocyanidin, and procyanidin were significantly increased their level in SG (Fig 4 and S8 Table). JA, which have been known as the most vital phytohormone to trigger plant defense against insect herbivores [2], was highly induced in RG, while only the hydroxylated derivatives of JA (12-OH-JA, tuberonic acid) was identified as DEMs in SG (Fig 4). In addition, when compared with SG, all the genes involved in jasmonic acid syntheses such as omega-3 fatty acid desaturase (*FAD7A-1*), linoleate 13S-lipoxygenase 2–1 (*LOX2*.*1*), and jasmonate O-methyltransferase (*JMT*) in RG were shown a higher expression level (S5 Table). Interestingly, the level of fructose was significantly repressed in RG, while it was significantly induced in SG during TG attack. We also observed that SG accumulated a significantly higher level of SA and theanine under either control or TG feeding condition (Fig 4).

## Discussion

It is imperative to use high-throughput omics techniques (e.g. transcriptomic, and metabolomic) based approach to identify key pathway metabolites and genes involved in TG defense in tea plants as follows in earlier studies for dissecting various biotic stresses [7–9]. In addition, different plant species or different ecotypes of the same species may trigger different defense responses to the same insect feeding. For example, the global transcriptional responses of two white cabbage cultivars differ remarkably in response to *P*. *rapae* feeding [25]. Therefore, elucidation of response mechanism and identification of key metabolites and genes involved in TG defense in RG could be a better approach to capture potential key candidates and control severe crop loss. Here, we describe a global comparison of the gene expression and metabolite profiles in resistant and susceptible genotypes of the tea plants following the challenge with the TG attack.

The sensing and intracellular transduction of feeding signals into appropriate downstream responses are essential for the adaptation and survival of plants. Oral secretions such as FACs and *β*-glucosidase from feeding insects can lead to a conformational change in plant cell-surface receptors, evoking an immediate and quick activation of downstream defense signalling pathways e.g., LRR-RLKs, Ser/Thr kinase, CDPKs, and MAPKs pathways [1,25,26]. PPI network analysis of DEGs which shown a higher expression level in RG was conducted to reaffirm key genes modulating TG defense in tea. Being in the PPI network hub and regulating more than 10 other genes, 39 TFs or PKs could thus be designated as key putative regulatory genes involved in TG defense in tea plants. Most of these candidate genes (e.g., *MYB308*, *WRKY41/75*, *MAPKKK19*, *CDPK2*, and *MYC4*) have been known to impart biotic stress resistance in several species [25–29].

GO enrichment of phosphorylation and cell surface receptor signaling pathway further highlight a vital role of PKs in RG during TG feeding. Among the 39 key candidate genes, 33 genes were belonging to LRR-RLKs, Ser/Thr kinase, CDPKs, and MAPKs. Upon herbivore feeding, the cytosolic Ca^2+^ increases dramatically, which in turn activates the calcium-binding sensory proteins such as calmodulin (CaMs), calmodulin-binding proteins, and CDPKs that promote transcriptional and phosphorylation signaling events [27]. CDPKs mediated plant defense signaling pathway has been reported to be significantly activated after feeding by *M*. *persicae* in *Arabidopsis* and *D*. *noxia* in wheat [28]. Tobacco CPK (NtCDPK2) has been shown to involved in the biosynthesis of JA and ethylene and, moreover, in cross-talk with the MAPK cascade activated by biotic stress [29]. MAPKKK19 to MKK9 pathway was identified as an important signal transduction branch in the *B*. *napus* defense responses to biotic stress [30]. In addition, several lines of evidence suggest that RLK/Pelle gene family tends to be enriched in genes that are highly responsive to biotic stress [31,32]. Recently, it has been reported that CDPK2, MPK2, MPK3, MPK6 and most of (7 out of 10) LRR-RLK genes were up-regulated in response to the TG feeding in tea plant [9]. The further activation of these PKs (e.g., CDPK2 and MPK3/HOG1) during TG feeding may facilitate the activation of downstream defense signaling genes in RG, thereby leading to an enhanced tolerance in TG stressed plants. In addition, these candidate PKs were found to connect with 282 other genes in the network, which may confer broad-spectrum insect resistance in plants. Therefore, transferring these PKs that can perceive a TG threat efficiently from RG to another and increase the level of resistance in SG or other species against TG.

Transcription factors disclose various genes like *MYB308/108*, *WRKY41/75*, *NAC062*, and *MYC4* present in network regulating other genes, and thus might be playing a crucial role in TG defense. Among these, NAC, MYB, and WRKY were also found in regulating various secondary metabolites biosynthesis (e.g., caffeine, theanine, and flavonoid) in tea plants. In Antirrhinum, the MYB308 has been reported to regulate flavonoids and lignin biosynthesis in transgenic tobacco [33]. MYC4 is a JAZ-interacting transcription factor, which known to mediate a subset of JA regulated signaling pathway [34]. WRKY75 controls crosstalk between SA and JA signal pathways, which are required to fine-tune of plant responses to pathogen and herbivore insects [35,36]. Similarly, in a recent study, several MYC and WRKY family members were also found to be induced after TG feeding in tea plants [9]. Hence, the expression level of these TFs may be tailored in response to TG damage and exhibited a higher expression level in RG either directly or indirectly via hormone signaling and secondary metabolite biosynthesis. The PPI analysis predicted 39 master regulators, thus can be considered as good candidates for enhancing tea plants’ resistance to TG attack.

SA and JA were two pivotal signaling molecules that involved in major plant defense mechanisms. It is a widely held view that SA and JA act antagonistically and induced in distinct patterns by pathogen and insect damage [2]. Cross-talk between signal transduction pathways triggered by these two hormones is thought to enable the plant to fine-tune pathogen and herbivore damage responses [37]. Accumulation of JA has been associated with insect resistance in *A*. *thaliana* and other plant species [36–38]. During TG attack, all the JA synthesis-related genes such as *FAD7A-1*, *LOX2*.*1*, and *JMT* in RG displayed significantly higher expression level compared to the SG [39,40]. Accordingly, the JA concentration is only found to significantly accumulate in RG. While SA, which suppresses some JA-regulated plant defenses, showed a constantly higher level in SG and could account for the observed suppression of JA signaling. In addition, the hydroxylated derivatives of JA (i.e., 12-OH-JA) may deactivate JA-dependent defense genes were also significantly induced in SG [41,42]. Hence, it could conceivably be hypothesized that the constitutively present of SA in SG might suppress JA signaling and thus misdirect tea plants against TG.

Recently, it became clear that JA could act as an elicitor to induce pathways synthesizing multiple branches of the most pivotal secondary metabolites (i.e. flavonoids, alkaloids, and terpenoids) with a wide structural variety in plant tissues [43]. Terpenoids are important members of the class of HIPVs, which serve as repellents or function in indirect defense by attracting natural enemies of the herbivorous insects [3]. Prior studies have shown that JA act as master switches for terpenoids formation by activating TPS genes in response to herbivore attack [44,45]. In the present study, the expression level of genes involved in diterpenoid, sesquiterpenoid, and triterpenoid biosynthesis pathway differed significantly between two cultivars, and most of the (7 out of 10) genes encoding terpene synthases such as *TPS03*, *TPS04* and *TPS21* displayed a higher expression level in RG. In summary, TPS genes might be more efficiently induced followed by activation of JA signaling cascade in RG for production of terpenoids in response to TG feeding.

Flavonoids that include aurones, flavan, flavones, flavonols, proanthocyanidins, dihydroflavonols, flavanones, chalcones, and anthocyanins play a central role in plant-environment interactions [46]. The accumulation of flavonoids have been reported to defend plants against various abiotic and biotic stresses such as herbivory insect, pathogens, and low temperature [46]. In our study, the expression level of genes related to the phenylpropanoid, anthocyanin, flavone and flavonol biosynthesis altered remarkedly when compare RG with SG during TG attack. All the *PAL* and *LDOX* were observed to show a higher expression level in RG. In plants, PAL is known to be a principal enzyme involved in a rate-limiting step of flavonoids biosynthesis, and supply the precursors for phytoalexins, protectants, lignin, and flavonoids [47]. Qualitative and quantitative alterations in these metabolites are known to play a vital role in resistance to insect pests. LDOX is a key regulated enzyme for anthocyanin biosynthesis, which was induced in response to various biotic and abiotic stresses, such as insect attacks, high-intensity light, UV light, high/low temperature, wounding, and drought [48,49]. For example, LDOX was reported to be strongly induced by *S*. *littoralis* herbivory in *A*. *thaliana*. In the present study, the observed accumulation of flavonoids seems to be a common mechanism operative in both cultivars to defense against TG attack. However, it was also known that the flavonoids produced varies according to the plant and insect species. In *C*. *sativa*, the major flavonoid in response to flea beetle feeding was quercetin glycoside, however, it has no defense effects against armyworm feeding in bertha [50]. In *A*. *thaliana*, accumulation of flavonol and anthocyanin enhanced the resistance to caterpillars, but it did not play a role in defense against aphid [51]. As expected, the metabolites involved in these pathways also showed different levels. The RG shown a significant change in catechins (C, GC, and EC), while SG shown a significant change in kaempferol, delphinidin, quercetin, proanthocyanidin, and procyanidin. Catechins, belonging to flavan-3-ol derivatives, are astringent bitter polyphenols and have been known to act as feeding deterrents against various insect pests. For instance, catechin and gallocatechin in leaves of *Q*. *robur* were identified to inhibit winter moth larvae, *O*. *brumata* [52]. In summary, the *PAL* and *LDOX* could promote the accumulation of catechins and anthocyanin in RG, which can act as feeding deterrents to affect the growth and development of TG.

To meet their nutritional requirements, herbivore insects tend to locate a healthy host plant that can provide them with suitable food (i.e., amino acids and sugars), and the ability to recognize toxic secondary metabolites is essential for their survival [53,54]. In this study, the remarkable alteration of carbohydrate and nitrogen metabolism (e.g., response to nutrient, cellular response to organonitrogen compound, hexose metabolic process, and carbohydrate catabolic and transport process) between two genotypes might lead to differing effects on TG feeding behavior. It is noteworthy that SG accumulated a significantly higher level of fructose and theanine than RG during TG attack. Fructose is a bona fide energy source for insects, which may act as a great feeding stimulant for TG. Theanine is one of the most abundant free amino acids in tea leaves; This compound was not only known to impose tea infusion an umami flavor but also have various benefits for human health [55]. According to these observations, we can infer that secondary metabolites such as flavonoids and terpenoids mentioned above can constitute the chemical barrier on TG feeding behavior in RG, whereas, a higher level of fructose and theanine accumulated in SG, can act as feeding stimulants.

## Conclusions

This study, for the first time performed a comparative transcriptome and metabolome profiling of the defense response to TG attack in resistant and susceptible tea plant genotypes. A large discrepancy in the molecular and chemical pattern between RG and SG during TG attack was detected. To defense against TG feeding, activation of cell surface and intracellular signaling pathway enable plants to perceive TG threat more efficiently. TFs are activated in the plant to regulate the TG defense either directly or indirectly via hormone signaling and secondary metabolite. JA was highly induced and could act as an elicitor to further activate pathways synthesizing defense-related secondary metabolites such as terpenoids and catechins, which can constitute the chemical barrier on TG feeding behavior. However, the constitutively present of SA might suppress JA signaling pathway and thus misdirect plants against TG. In addition, higher accumulation of fructose and theanine in plants can act as feeding stimulants when facing TG attack. Furthermore, 39 putative key defense-related TFs or PKs identified by PPI network analysis will enable the breeding of tea cultivars with enhanced resistance to insect herbivores and deserve further systematic functional validation.

## Supporting information

S1 Fig GO and KEGG annotation and classification of *C*. *sinensis* transcriptome. The functional category distribution of 342,961 unigenes in (A) GO and (B) KEGG databases.
(TIF)

S2 Fig Heatmap of pairwise Pearson correlation between samples. The color of the heatmap indicates the positive correlation of gene expression between samples.
(TIF)

S3 Fig qRT-PCR validation for 6 DEGs. Error bars indicate standard error of the mean expression values from three biological replicates.
(TIF)

S4 Fig Distribution of samples clustered using PCA. Sample replicates from (A) LC-MS and (B) GC-MS were grouped in ellipses.
(TIF)

S1 Table Sequences of primers used for the qRT-PCR assay. (XLSX)

S2 Table Quality of sequencing. (DOCX)

S3 Table Length distribution of assembled transcripts and unigenes. (DOCX)

S4 Table Summary for the annotation of unigenes. (DOCX)

S5 Table List of terpene synthases, JA and flavonoids biosynthesis related genes. (XLSX)

S6 Table GO enrichment of up-regulated genes. (XLSX)

S7 Table Potential candidates for enhancing plant resistance to TG. (XLSX)

S8 Table List of differentially expressed metabolites. (XLSX)
