# Supplementary figures and images for: Transcriptome and metabolome analysis reveal candidate genes and biochemicals involved in tea geometrid defense in Camellia sinensis
Source: PLoS One. 2018 Aug 1;13(8):e0201670. doi: 10.1371/journal.pone.0201670 (PMC6070272; doi:10.1371/journal.pone.0201670)

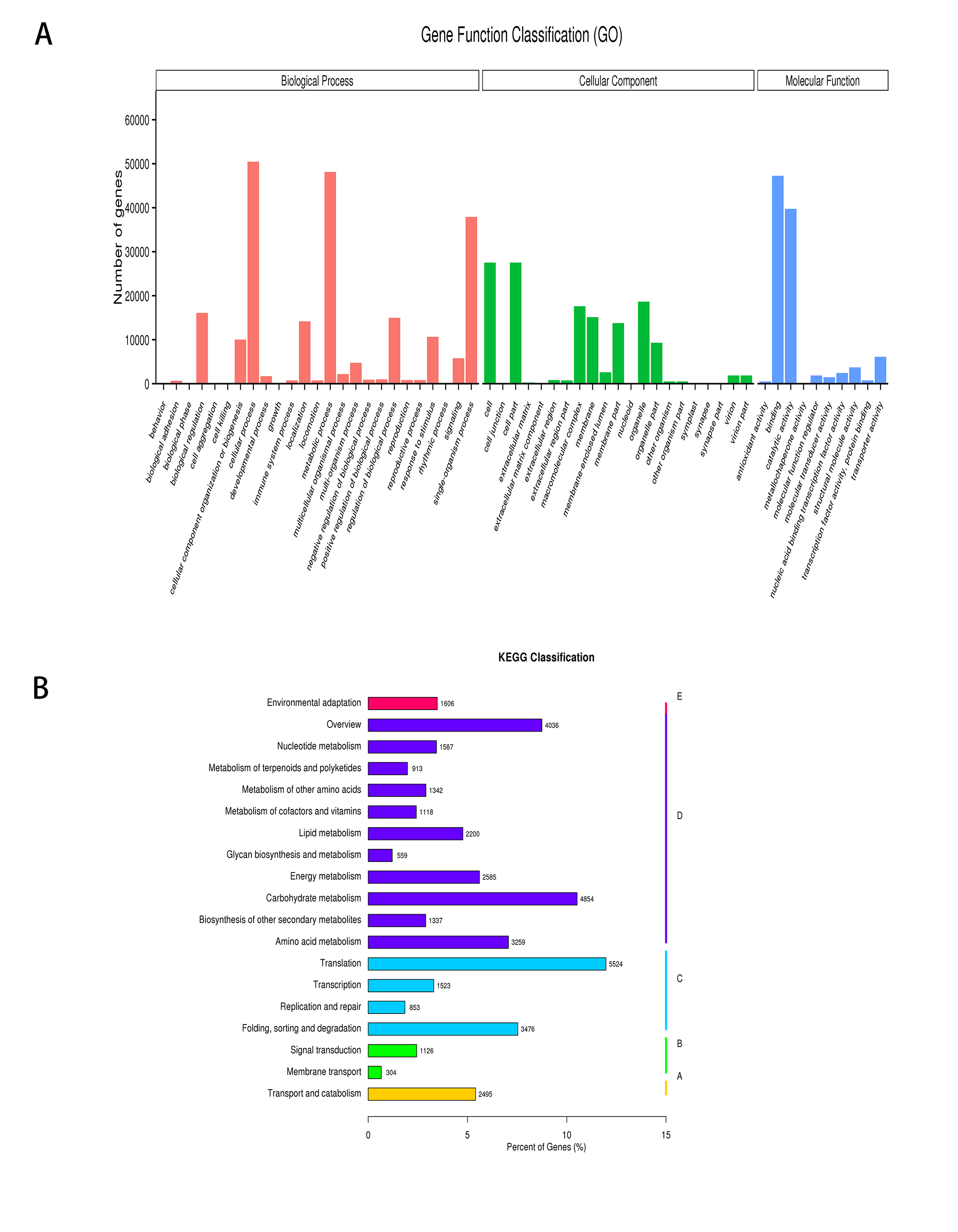

Supplement: S1 Fig — The functional category distribution of 342,961 unigenes in (A) GO and (B) KEGG databases. (TIF) [file pone.0201670.s001.tif]

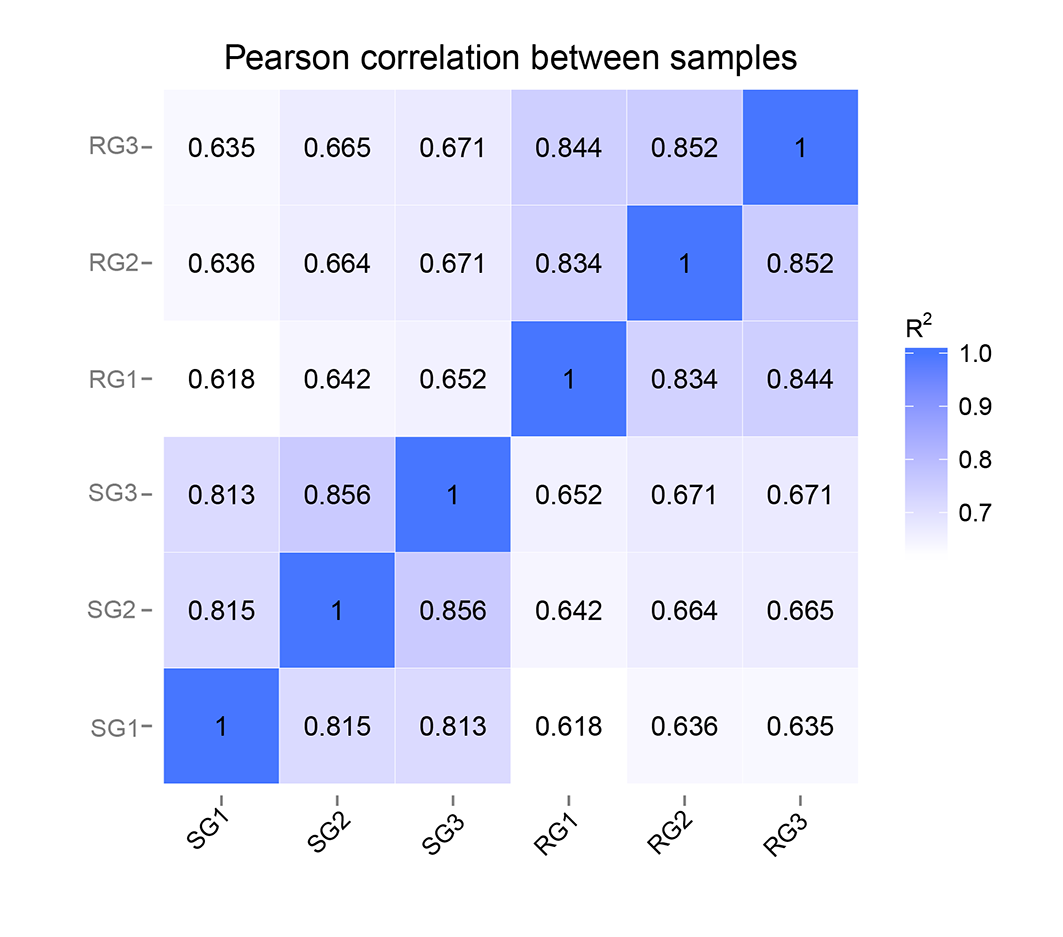

Supplement: S2 Fig — The color of the heatmap indicates the positive correlation of gene expression between samples. (TIF) [file pone.0201670.s002.tif]

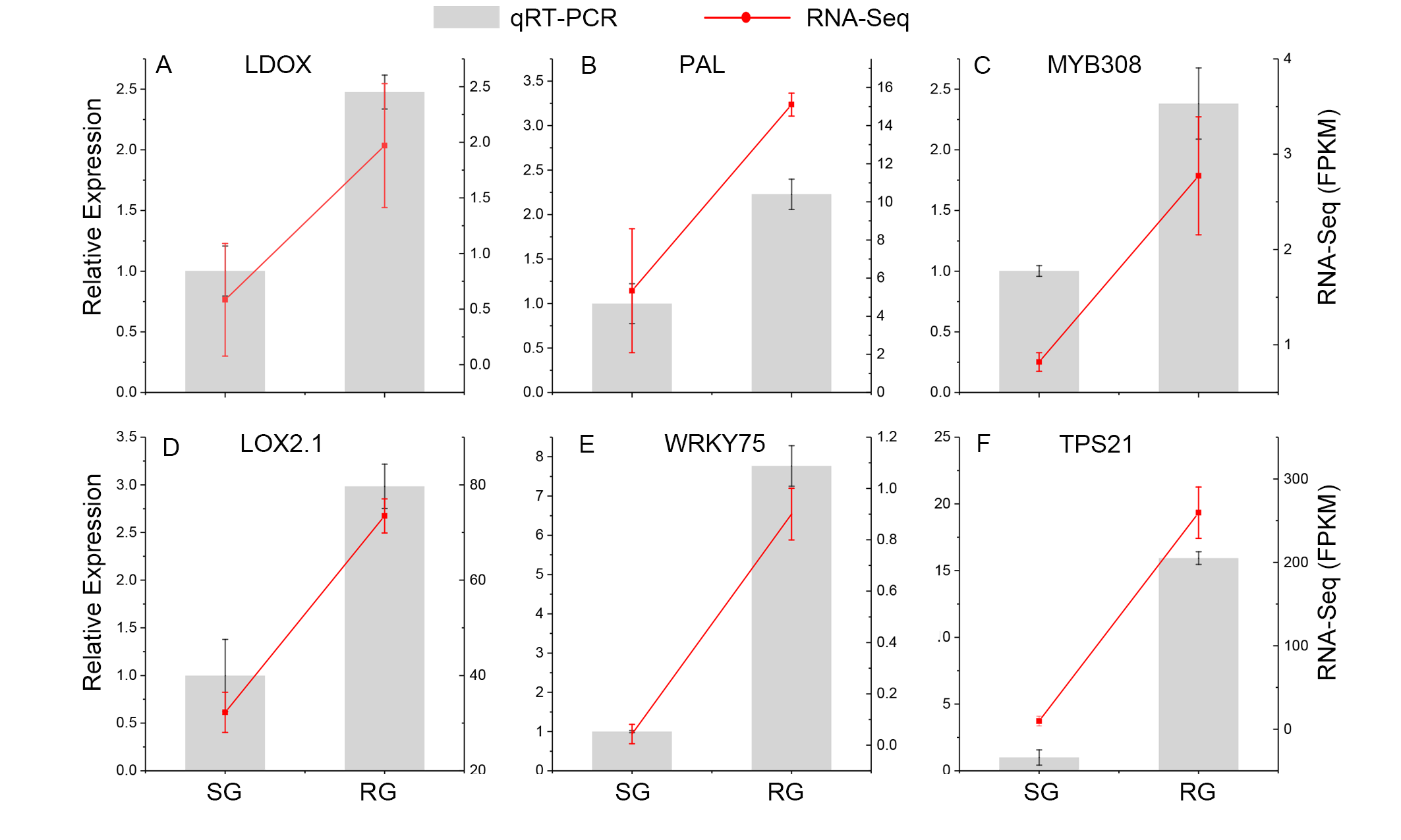

Supplement: S3 Fig — Error bars indicate standard error of the mean expression values from three biological replicates. (TIF) [file pone.0201670.s003.tif]

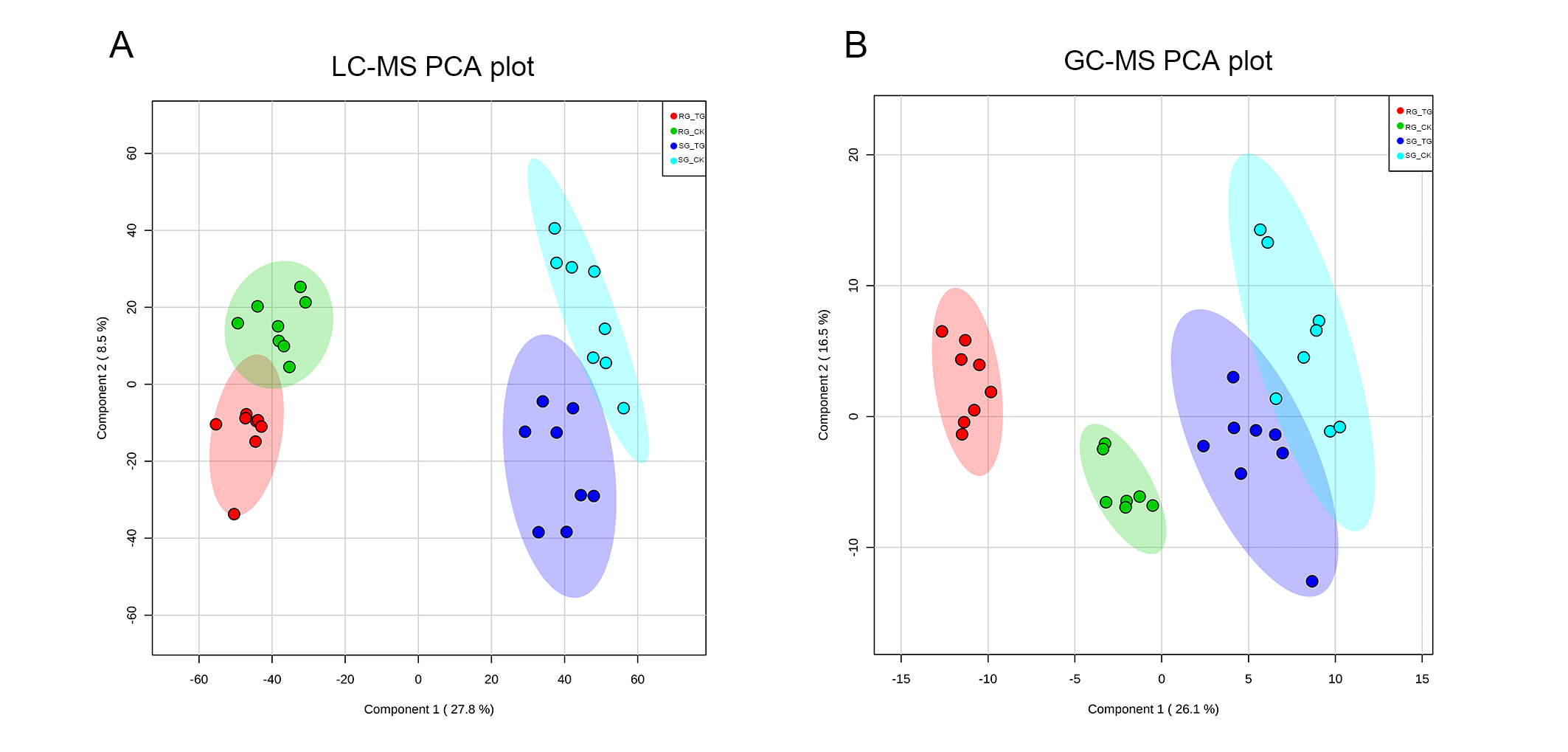

Supplement: S4 Fig — Sample replicates from (A) LC-MS and (B) GC-MS were grouped in ellipses. (TIF) [file pone.0201670.s004.tif]
