# Supplementary material for: Transcriptome and metabolome analysis reveal candidate genes and biochemicals involved in tea geometrid defense in Camellia sinensis
Source: PLoS One. 2018 Aug 1;13(8):e0201670. doi: 10.1371/journal.pone.0201670 (PMC6070272; doi:10.1371/journal.pone.0201670)
Supplement: S2 Table — (DOCX) [file pone.0201670.s006.docx]

**S2 Table. Quality of sequencing.**

| **Sample** | **Raw Reads** | **Clean reads** | **Clean bases** | **Error (%)** | **Q20 (%)** | **Q30 (%)** | **GC (%)** |
| --- | --- | --- | --- | --- | --- | --- | --- |
| Cs_P1 | 130,285,750 | 125,522,556 | 18.83G | 0.01 | 97.1 | 92.95 | 44.5 |
| Cs_P2 | 110,535,262 | 106,411,348 | 15.96G | 0.01 | 97.01 | 92.75 | 45.14 |
| Cs_P3 | 120,048,424 | 115,632,210 | 17.34G | 0.01 | 97.1 | 92.9 | 44.92 |
| Cs_M1 | 126,154,652 | 121,875,604 | 18.28G | 0.01 | 97.02 | 92.72 | 44.65 |
| Cs_M2 | 126,609,138 | 122,295,100 | 18.34G | 0.01 | 97.03 | 92.75 | 44.86 |
| Cs_M3 | 117,045,228 | 113,061,274 | 16.96G | 0.01 | 97.18 | 93.03 | 44.73 |
