# Supplementary material for: Transcriptome and metabolome analysis reveal candidate genes and biochemicals involved in tea geometrid defense in Camellia sinensis
Source: PLoS One. 2018 Aug 1;13(8):e0201670. doi: 10.1371/journal.pone.0201670 (PMC6070272; doi:10.1371/journal.pone.0201670)
Supplement: S3 Table — (DOCX) [file pone.0201670.s007.docx]

**S3 Table. Length distribution of assembled transcripts and unigenes.**

| **Transcript length interval** | **Number of transcripts** | **Number of unigenes** |
| --- | --- | --- |
| **200-500bp** | 299,424 | 228,981 |
| **500-1kbp** | 121,166 | 65,319 |
| **1k-2kbp** | 83,462 | 34,087 |
| **>2kbp** | 39,631 | 14,574 |
| **Total** | 543,683 | 342,961 |
| **Min Length** | 201 | 201 |
| **Mean Length** | 755 | 594 |
| **Median Length** | 441 | 352 |
| **Max Length** | 15,022 | 15,022 |
| **N50** | 1,197 | 834 |
