# Supplementary material for: Transcriptome and metabolome analysis reveal candidate genes and biochemicals involved in tea geometrid defense in Camellia sinensis
Source: PLoS One. 2018 Aug 1;13(8):e0201670. doi: 10.1371/journal.pone.0201670 (PMC6070272; doi:10.1371/journal.pone.0201670)
Supplement: S4 Table — (DOCX) [file pone.0201670.s008.docx]

**S4 Table. Summary for the annotation of unigenes.**

|  | **Number of Unigenes** | **Percentage (%)** |
| --- | --- | --- |
| **Annotated in NR** | 121,628 | 35.46 |
| **Annotated in NT** | 74,024 | 21.58 |
| **Annotated in KO** | 46,137 | 13.45 |
| **Annotated in Swiss-Prot** | 95,872 | 27.95 |
| **Annotated in PFAM** | 91,533 | 26.68 |
| **Annotated in GO** | 93,667 | 27.31 |
| **Annotated in KOG** | 51,793 | 15.1 |
| **Annotated in all Databases** | 18,456 | 5.38 |
| **Annotated in at least one Database** | 156,440 | 45.61 |
| **Total Unigenes** | 342,961 | 100 |
